# Wegener's Granulomatosis presenting with an abscess in the parotid gland: a case report

**DOI:** 10.1186/1752-1947-3-19

**Published:** 2009-01-23

**Authors:** Marcel Geyer, Gautham Kulamarva, Anne Davis

**Affiliations:** 1 Department of Otorhinolaryngology, Head and Neck Surgery, Queen Alexandra Hospital, Southwick Hill Road, Cosham, Portsmouth, Hampshire, UK

## Abstract

**Introduction:**

Wegener's Granulomatosis is a vasculitis of uncertain aetiology. Affected patients usually present with disease of the respiratory and renal tracts. Classic symptoms and clinical findings, together with serology titres positive for anti-neutrophil cytolplasmic antibody against proteinase 3 confirm the diagnosis. Wegener's Granulomatosis can occasionally involve other organs, but solitary parotid gland disease is uncommon; patients generally also have systemic disease.

**Case Presentation:**

We report a case of Wegener's Granulomatosis in a 69-year-old Caucasian female presenting initially with an isolated parotid abscess and only subsequently developing nasal, paranasal sinus and respiratory symptoms. We describe the clinical course, diagnostic difficulties, imaging and histopathology of this case.

**Conclusion:**

Major salivary gland infection is not an uncommon ENT disorder, but the clinician should be wary of the patient who fails to respond appropriately to adequate therapy. In such cases a differential diagnosis of Wegener's Granulomatosis should be considered, as early recognition and treatment of this potentially fatal disease is paramount.

## Introduction

Heinz Klinger was first to describe the disease process of Wegener's Granulomatosis (WG) in 1932.[1]. Subsequently, Frederick Wegener published his two papers in 1936 [2] and 1939 [3] describing post-mortem studies of two patients who died of disseminated vasculitis. WG usually presents as a triad of airway necrotising granulomas, systemic vasculitis and focal necrotising glomerulonephritis. The diagnosis of WG is based on clinical findings and positive anti-neutrophil cytolplasmic antibody against proteinase 3 (cANCA-PR3) serology. A biopsy is rarely histologically diagnostic [4]. Our case is unusual in that the patient presented initially solely with a parotid abscess in the absence of typical signs or symptoms of rhinologic or systemic WG.

## Case Presentation

A 69-year-old Caucasian female presented with a 10-day history of worsening pain and swelling over the region of the left parotid gland. She had been unsuccessfully treated with a 7-day course of oral penicillin by her general practitioner, but with no improvement. She denied any precipitating cause, though she had been feeling 'under the weather' and had lost her appetite over some weeks; there was no history of parotid disease. There was no other significant medical history and she was a non-smoker.

Clinically she was pyrexial (37.7°C), dehydrated and in discomfort due to left facial swelling with a marked degree of trismus. An 8 cm by 5 cm tense, tender, fluctuant swelling was palpable in the left parotid gland, and the lower pole of the pinna was displaced laterally. Examination of neck, ears and nose (by flexible nasendoscopy) was normal. Intra-oral inspection confirmed that there was no discharge from Stenson's duct and no calculus was palpable. She did however demonstrate a House-Brackmann Grade II palsy of the left marginal mandibular nerve; all other cranial nerves were intact. A full blood count showed a neutrophilia of 15.0 × 10^9^/l (WCC = 17.9 × 10^9^/l); plasma C-reactive protein (CRP) was markedly raised (285 mg/l). An initial chest X-ray was normal.

**Figure 1:**
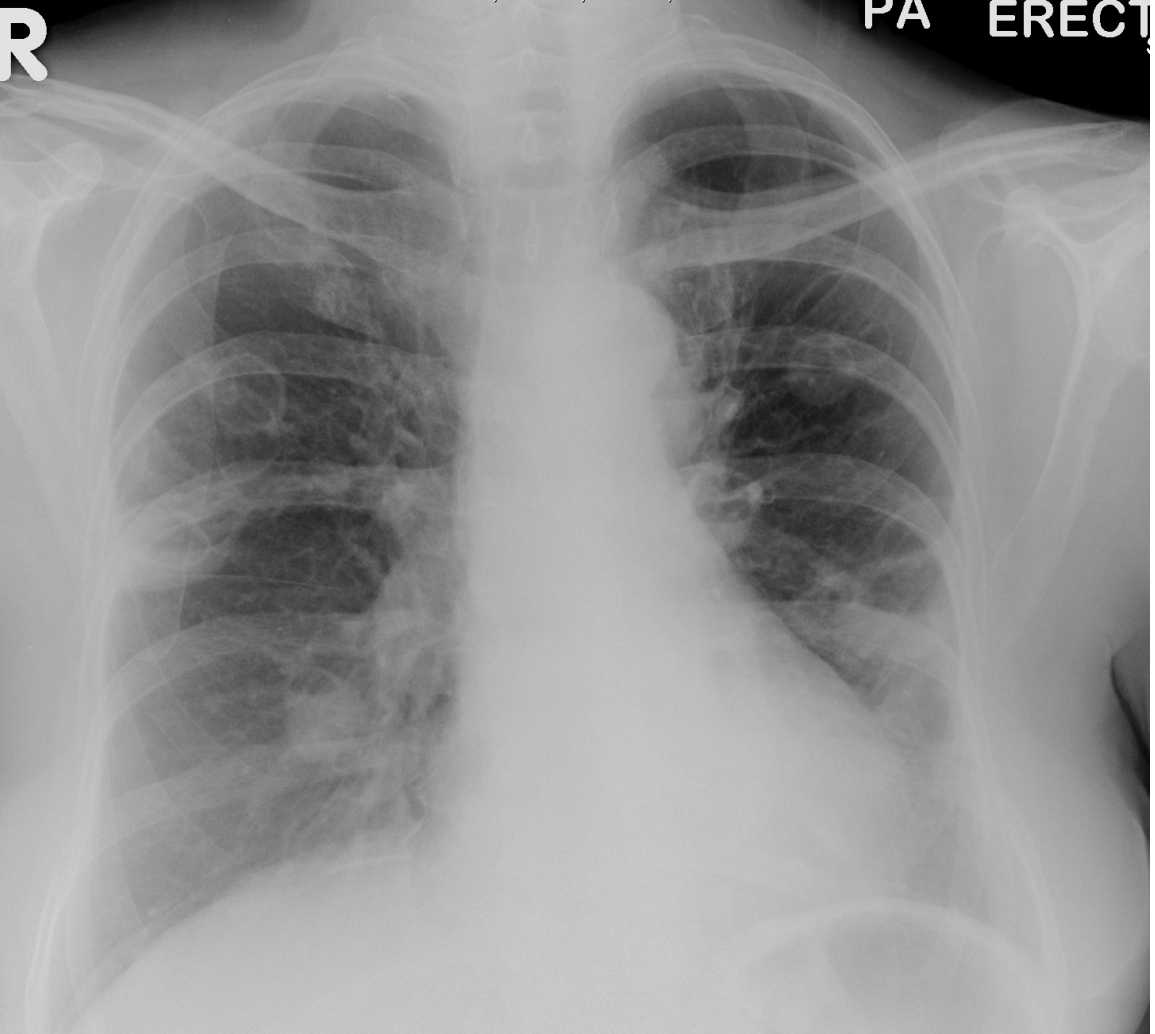
**X-ray Chest – Cavitating pneumonia**.

Large-bore needle aspiration of 15 ml of frank pus from the left parotid gland provided some relief and lessened the trismus. Intravenous antibiotic treatment with Metronidazole and Amoxicillin/Clavulanate and rehydration were commenced. An ultrasound scan the following day could not identify a collection in the left parotid. However, as the patient remained unwell, formal incision and drainage was performed after 3 days. A further 10 ml of pus was drained and a biopsy taken which showed non-specific inflammation. Despite this treatment, within a week her condition deteriorated, complicated by respiratory symptoms (chest pain, dyspnoea and a non-productive cough). A repeat chest X-ray showed fixed infiltrates and cavitation of both lung fields (Figure 1). Transfer of care to the respiratory team and empirical treatment for suspected Staphylococcus Aureus cavitating pneumonia led to improvement and discharge home 17 days after admission.

Unfortunately she was re-admitted after a further 5 days with progressive respiratory failure requiring transfer to the Intensive Care Unit (ICU) and ventilatory support. Ear, nose and throat (ENT) examination in ICU revealed bilateral otitis externa, destruction of the nasal septum and granulomatous appearance of the mucosa. An urgent CT scan showed extensive septal and lateral nasal wall destruction without intracranial complications.

**Figure 2:**
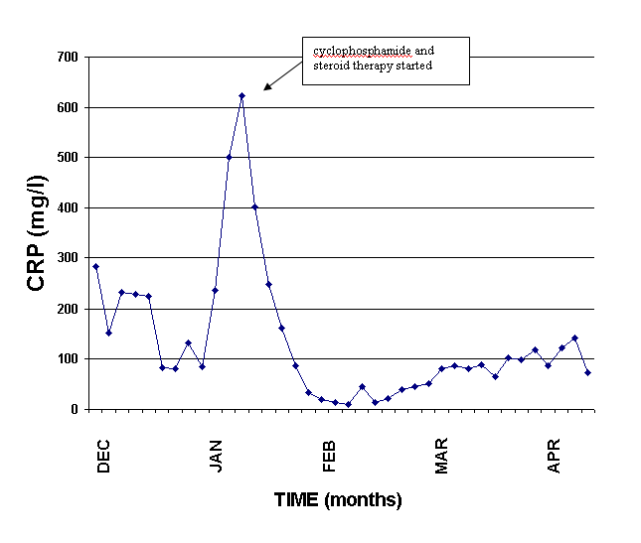
**Patient serial serum CRP measurement**.

The combined evidence of a history of feeling unwell with loss of appetite, clinical findings of septal perforation and friable nasal mucosa as well as the radiological features of pulmonary infiltrates and cavitation suggested WG. A serum cANCA titre was strongly positive (Ratio 2.5 of Proteinase 3), confirming the diagnosis. Treatment with Methylprednisolone, Prednisolone and Cyclophosphamide was given and adjunctive supportive measures continued, leading to clinical improvement and gradual disease resolution. The patient was ultimately discharged 3 months after the original admission. Serial CRP measurements correlated well with the disease severity (Figure 2). Histological examination of a nasal biopsy taken in ICU confirmed features of WG retrospectively: necrotising granulomata, foci of necrosis and blood vessels showing fibrinoid necrosis and inflammation of their walls. Neither acid-fast bacilli nor evidence of malignancy were found.

## Discussion

The often rapidly progressive and potentially fatal disease Wegener's Granulomatosis affects mainly the upper and lower respiratory tracts and the kidneys. Early recognition and treatment is paramount in preventing severe organ damage. The peak age incidence is at 50 to 60 years and confined almost entirely to Caucasoid individuals [5,6]. The exact aetiology remains unclear. It may represent some form of hypersensitivity reaction and immune response to an unknown stimulus. Two types of WG are described: the most common is a multi-system disease; the other is confined to one area of the respiratory tract. Non-specific systemic symptoms of WG include fatigue, fever, arthralgia and weight loss. Head and neck symptoms occur in 90% of patients: the nose, paranasal sinuses (up to 80%) [6] and middle ear [7] are commonly affected. Nasal symptoms of the disease include serosanguinous discharge and headache and pain over the dorsum. Signs comprise crusts covering friable mucosa, ulceration, septal perforation and saddle-nose deformity. Oropharyngeal, laryngeal and facial nerve involvement, among others, has been described. Involvement of salivary glands occurs in less than 1% [8].

The largest series described five cases of WG involving the salivary glands [9]. Disease of a major salivary gland almost always coincides with other head and neck or pulmonary signs and symptoms [10]. Thus major salivary gland involvement is a rare presentation of WG, and all patients described have had concomitant nasal, ear or lung symptoms and signs [10-15]. One patient went on to develop a parotid abscess following admission [12]. None have presented *ab initio *with an abscess but without other symptoms, as we describe here.

The diagnosis is based on clinical criteria including oral ulcers and nasal serosanguinous discharge, abnormal urinalysis and chest X-ray, supported by the histological findings of an adequate biopsy showing granulomatous inflammation and positive laboratory studies (cANCA-PR3 titres). Therapeutic response to immunosuppressive agents (cyclophosphamide or azothioprine) combined with steroids is good, with remission rates of up to 90%. If treatment is initiated early, involvement of the lower respiratory tract and kidneys may be avoided. Sinonasal manifestations may be treated medically with saline douching and topical nasal or systemic steroids. For bacterial superinfection antibiotics are prescribed. Long term follow up is essential to detect possible relapse, suggested by rising ANCA levels.

## Conclusion

Major salivary gland infection is not an uncommon ENT disorder, but the clinician should be wary of the patient who fails to respond appropriately to adequate therapy. In such cases a differential diagnosis of WG should be considered, as early, appropriate treatment is paramount in preventing significant morbidity or mortality.

## Abbreviations

CRP: C reactive protein; cANCA PR3: anti-neutrophil cytoplasmic antibody against proteinase 3; WCC: white cell count; ENT: ear, nose and throat

## Competing interests

The authors declare that they have no competing interests.

## Authors' contributions

MG was a major contributor in writing the manuscript. GK reviewed the patient's notes, collected the haematological and histological data and radiology slides and

AD was a major contributor in writing the manuscript. All authors read and approved the final manuscript.

## Consent

Written informed consent was obtained from the patient for publication of this case report and accompanying images. A copy of the written consent is available for review by the Editor-in-Chief of this journal.

## References

[B1] Klinger H Grenzformen der Periarteritis Nodosa Fr Z Pathol 1931 42 455 480

[B2] Wegener F Über generalisierte, septische Gefäßerkrankungen Verh Dtsch Ges Pathol 1936 29 202 210

[B3] Wegener F Über eine eigenartige rhinogene Granulomatose mit besonderer Beteiligung des Arteriensysytems und der Nieren Beitr Path Anat 1939 102 36 38

[B4] Langford CA Fauci AS Fauci AS, Braunwald E, Kasper DL, Hauser SL, Longo DL, Jameson JL, Loscalzo J The vasculitis Syndromes. Harrison's Principles of Internal Medicine 2001 15 New York: The McGraw-Hill companies Inc 1937 1939

[B5] Saravanappa N Bibas A Singhal A Davis JP Unilateral parotid swelling as initial manifestation of Wegener's granulomatosis J Otolaryngol 2006 396 397 11770152

[B6] Murty GE Wegener's granulomatosis: Otorhinological manifestations Clin Otorlaryngol 1990 15 385 393 10.1111/j.1365-2273.1990.tb00488.x 2225511

[B7] Kempf HG Ear involvement in Wegener's granulomatosis Clin Otolaryngol 1989 5 451 456 10.1111/j.1365-2273.1989.tb00403.x 2582643

[B8] Lloyd G Lund VJ Beale T Howard D Rhinologic Changes in Wegener's Granulomatosis J Laryngol Otol 2002 116 565 569 12238684 10.1258/002221502760132737

[B9] Specks U Colby TV Olsen KD DeRemee RA Salivary gland involvement in Wegener's Granulomatosis Arch Otolaryngol Head Neck Surg 1991 2 218 223 1991069 10.1001/archotol.1991.01870140106018

[B10] Chegar BE Kelly RT Wegener's Granulomatosis presenting as unilateral parotid swelling Laryngoscope 2004 114 1730 1733 10.1097/00005537-200410000-00010 15454762

[B11] Bülbül Y Ozlü T Oztuna F Wegener's granulomatosis with parotid gland involvement and pneumothorax Med Princ Pract 2003 12 133 137 10.1159/000069111 12634471

[B12] Imamoglu M Bahadir O Reis A Parotid gland involvement as an initial presentation of Wegener's granulomatosis Otolaryngol Head Neck Surg 2003 4 451 453 10.1016/S0194-5998(03)00626-0 14574306

[B13] Murty GE Mains BT Bennett MK Salivary gland involvement in Wegener's granulomatosis J Laryngol Otol 1990 3 259 261 2341787 10.1017/s0022215100112435

[B14] Benson-Mitchell R Tolley N Croft CB Roberts D Wegener's granuloma – presenting as a unilateral parotid swelling J Laryngol Otol 1994 108 431 432 8035127 10.1017/s0022215100126994

[B15] Liu SY Vlantis AC Lee WC Bilateral parotid and Submandibular Enlargement: A rare feature of Wegener's Granulomatosis J Laryngol Otol 2003 117 148 150 12625895 10.1258/002221503762624666

